# Degranulating mast cells in fibrotic regions of human tumors and evidence that mast cell heparin interferes with the growth of tumor cells through a mechanism involving fibroblasts

**DOI:** 10.1186/1471-2407-5-121

**Published:** 2005-09-21

**Authors:** Michael Samoszuk, Emi Kanakubo, John K Chan

**Affiliations:** 1 Department of Pathology and Laboratory Medicine, University of California, Irvine, California USA; 2 Department of Obstetrics and Gynecology, Stanford University, Stanford, California USA

## Abstract

**Background:**

The purpose of this study was to test the hypothesis that mast cells that are present in fibrotic regions of cancer can suppress the growth of tumor cells through an indirect mechanism involving peri-tumoral fibroblasts.

**Methods:**

We first immunostained a wide variety of human cancers for the presence of degranulated mast cells. In a subsequent series of controlled in vitro experiments, we then co-cultured UACC-812 human breast cancer cells with normal fibroblasts in the presence or absence of different combinations and doses of mast cell tryptase, mast cell heparin, a lysate of the human mast cell line HMC-1, and fibroblast growth factor-7 (FGF-7), a powerful, heparin-binding growth factor for breast epithelial cells.

**Results:**

Degranulating mast cells were localized predominantly in the fibrous tissue of every case of breast cancer, head and neck cancer, lung cancer, ovarian cancer, non-Hodgkin's lymphoma, and Hodgkin's disease that we examined. Mast cell tryptase and HMC-1 lysate had no significant effect on the clonogenic growth of cancer cells co-cultured with fibroblasts. By contrast, mast cell heparin at multiple doses significantly reduced the size and number of colonies of tumor cells co-cultured with fibroblasts, especially in the presence of FGF-7. Neither heparin nor FGF-7, individually or in combination, produced any significant effect on the clonogenic growth of breast cancer cells cultured without fibroblasts.

**Conclusion:**

Degranulating mast cells are restricted to peri-tumoral fibrous tissue, and mast cell heparin is a powerful inhibitor of clonogenic growth of tumor cells co-cultured with fibroblasts. These results may help to explain the well-known ability of heparin to inhibit the growth of primary and metastatic tumors.

## Background

In animal models of carcinogenesis, mast cells have been shown to play an important role in modulating angiogenesis, connective tissue remodeling, blood clotting, and the growth of tumors [1-3]. Similarly, a number of clinical investigators, including our laboratory, have documented the natural presence of mast cells in various human tumors, including breast cancer and melanoma [4-6]. In these studies, the mast cells were described as being predominantly located in the fibrous tissue stroma adjacent to the tumor cells rather than within the core of the tumor.

Collectively, these observations prompted us to speculate that the mast cells that are naturally present next to tumors might modulate the growth of the tumor cells through an indirect mechanism involving the fibroblasts that are adjacent to the tumor cells. It is now well-established, for example, that fibroblasts play a pivotal role in promoting the growth of many types of cancer cells [7-9] and that many mast cell granule components exert powerful effects on fibroblasts [2,10]. Therefore, in order to test this hypothesis, we first performed a comprehensive, immunohistochemical survey of a variety of human tumors to determine if mast cells are generally restricted to the fibrous tissue adjacent to tumor cells. To this end, tumor sections were immunostained for the presence of mast cell tryptase, a serine protease that is indicates the presence of intact and degranulated mast cells in tissues. This marker was selected instead of CD117 or other markers of intact mast cells because it can specifically identify intact mast cells as well as regions where mast cells have disintegrated and released the contents of their granules into the tissue. Because mast cell granules contain tryptase that is strongly bound to heparin [11,12], tryptase co-localizes with heparin in tissue sections and can therefore serve as an effective surrogate marker for the presence of heparin in tissues. This property is important because reliable immunohistochemical assays to detect the presence of mast cell heparin in tissue sections are generally not available.

In the second part of this study, we explored the effects of two major components of mast cell granules (tryptase and heparin) and FGF-7 on the clonogenic growth of a human breast cancer cell line co-cultured with normal human fibroblasts in a simple co-culture system that we have previously described in detail [13]. We specifically selected mast cell tryptase for study because it exerts profound effects on fibroblast function and proliferation [2,10]. Heparin, a highly sulfated proteoglycan produced exclusively by mast cells, was included in this study because our previous experiments with mice that were genetically or enzymatically depleted of mast cell heparin [5,14] have consistently demonstrated that tumors grow significantly faster in heparin-depleted mice than in control mice. FGF-7 was incorporated into our experiments because it is a powerful, heparin-binding growth factor for breast epithelial cells that is produced by fibroblasts [15-18]. Moreover, previous studies have clearly demonstrated that fibroblasts are the predominant source of FGF-7 in malignant breast tissues [18,19].

Here we demonstrate that degranulating mast cells are restricted almost exclusively to the peri-tumoral fibrous tissue in human cancers. Moreover, we show that heparin significantly interferes with the clonogenic growth of breast cancer cells co-cultured with fibroblasts in the presence or absence of exogenous FGF-7. These results lead us to conclude that heparin from mast cells can suppress the growth of tumor cells through an indirect mechanism involving the fibroblasts adjacent to the tumor.

## Methods

### Primary human tumor and normal tissue samples

After obtaining institutional review board approval for this project, we retrieved the most recent available paraffin blocks from the surgical pathology archives of UCI Medical Center (Orange, CA) and Long Beach Memorial Medical Center (Long Beach, CA) for the following types of tumors: breast cancer (n = 10); head and neck cancer (n = 10); lung cancer (n = 10), non-Hodgkin's lymphoma (n = 5); Hodgkin's lymphoma (n = 8); ovarian cancer (n = 10) and their normal tissue counterparts (n = 3 for each tumor type).

### Immunostaining for mast cell tryptase

Representative sections of each tumor and normal tissue counterpart were immunostained for mast cell tryptase using a procedure that has been previously described in detail [5,6] and also histochemically stained with Giemsa stain to highlight intact mast cells (identifiable by their dark purple granules). In brief, de-paraffinized tissue sections of each tumor and normal tissue were first subjected to antigen retrieval using microwave treatment in citrate buffer. The tissue sections were then incubated with a murine monoclonal antibody directed against human mast cell tryptase (Dako-Cytomation, Carpinteria, California USA) at a concentration of 10 micrograms/mL for 2 hours. Bound primary antibody was then detected using the Dako LSAB+ peroxidase kit-universal (Dako-Cytomation) in accordance with the manufacturer's directions. The positive control for this antibody was a section of human breast cancer with extensive infiltration by mast cells noted on routine histologic examination [6]. Because virtually all normal tissues contain at least a few mast cells, the negative controls were serial sections of the same tissues incubated with isotype-matched, non-immune IgG from a mouse. The specificity of the immunostaining was confirmed on two slides by competitive inhibition with human mast cell tryptase at a concentration of 1 microgram/mL (Sigma-Aldrich, St. Louis, Missouri, USA).

An experienced pathologist then counted the numbers of mast cells in three representative, non-overlapping, 40 × microscopic fields of the peripheral and central regions (core) of each tumor section. For the normal tissue counterparts, the numbers of mast cells were counted in three, randomly selected and non-overlapping 40 × microscopic fields of each slide. The means and standard deviations of mast cell numbers in the two tumor regions and in the normal tissue counterparts were then calculated and compared statistically, using a two-tailed, unpaired t-test for significance.

### Co-culture assay

The co-culture assay was performed as previously described [13] In brief, UACC-812 breast cancer cells (ATCC, Manassas, VA) were seeded at a density of 100 cells per well of a 96-well microtiter plate containing a confluent monolayer of allegedly normal human fibroblasts (CCD 1068 SK), also from ATCC. The allegedly normal human fibroblasts were derived from the skin of the breast from a woman who underwent a mastectomy for breast cancer. The cells were co-cultured in Eagles minimal essential medium (ATCC) containing 10% fetal calf serum (Sigma-Aldrich) and penicillin-streptomycin (ATCC) that was then supplemented on days 1, 4, 7, 10, and 13 after seeding with human mast cell tryptase derived from human lung (1, 5, or 10 μgrams/mL; Sigma-Aldrich, St. Louis, MO), or purified porcine intestinal derived heparin, high molecular weight type (0.1,1.0, 10.0 and 100 units/mL; Sigma-Aldrich); and/or human fibroblast growth factor-7 (100 ng/mL;Chemicon International, Temecula, CA). It should be noted that the lung-derived tryptase used in these experiments contained 0.05 mM heparin, but the final concentration of heparin in the tryptase solution used in our experiments was approximately 20-fold less than the lowest concentrations of porcine heparin used alone. On day 14, the cells were fixed in situ by incubating the wells with ice-cold methanol for 10 minutes. The cells were then stained in situ with 0.1% toluidine blue, and we used routine light microscopy to count the numbers of colonies of tumor cells containing 10 or more tumor cells in each well. Each assay was performed in triplicate, and the means and standard deviations for the numbers of colonies in each treatment and control group were then calculated and compared using a two-tailed unpaired t-test for significance.

The positive control for the assay consisted of identical numbers of tumor cells co-cultured on a monolayer of fibroblasts without any additional supplements to the growth medium. In order to determine if any effects that we observed were attributable to the fibroblasts, we also performed the clonogenic assay as described above, but in the absence of a monolayer of fibroblasts.

For comparative purposes, we also performed the co-culture assay in the presence of 50 microliters of lysate equivalent to 10^6 ^HMC-1 cells/mL. The HMC-1 cell line is a human mast cell line derived from a patient with mast cell leukemia [20] and was the generous gift of Dr. J.H. Butterfield from the Mayo Clinic (Rochester, Minnesota, USA). It expresses many of the markers and cytoplasmic components of normal mast cells [20] and was cultured under conditions that have been previously described [20]. The HMC-1 lysate was produced by a procedure described by Huttunen et al [21]. To confirm the presence of heparin in the HMC-1 cells, we stained cytopreparations of the cultured cells for 30 seconds with 0.1% toluidine blue, a metachromatic dye that binds strongly to heparin and produces an intense blue color in the granules of normal mast cells [11].

### Measurement of tumor colony size

Digital images of 10–50 representative colonies of tumor cells in each well were acquired at 40 × magnification with a Nikon Eclipse E600 microscope equipped with a Spot II digital camera (Diagnostic Instruments, Sterling Heights, MI). Because some treatments resulted in far fewer colonies (see below) than the control group, the numbers of colonies that were analyzed among the different treatment groups varied from group to group. The digital images were then analyzed using a digital tool (Image Pro Plus version 4.5, Media Cybernetics, Inc., Silver Spring, MD) to outline the area of the tumor cell colony in each image. The area in each colony was electronically integrated, and we plotted the distribution of areas as a frequency histogram for each treatment and control group. The data were then analyzed statistically using the Mann-Whitney U-test for two samples and ranked observations, not paired.

### Detection of FGF-7 in cultured human fibroblasts

A number of other investigators have previously documented with Western blotting and in situ hybridization that fibroblasts secrete FGF-7 and are the predominant source of FGF-7 in normal tissues and in breast cancers [18,19]. In order to confirm that the monolayer of fibroblasts that was used in our experiments was also producing FGF-7, we first immunostained the fibroblasts in situ with a monoclonal antibody directed against FGF-7 (Chemicon International), using a procedure that we have previously described in detail [13]. The negative controls consisted of an irrelevant monoclonal antibody or blocking buffer alone. As an additional confirmation for the production of FGF-7 by the fibroblasts, we analyzed the gene expression profile of the cultured fibroblasts, using a DNA microarray as previously described by us [13].

In order to provide additional confirmation of the presence of FGF-7 in the fibroblasts, we performed Western blotting of an immunoprecipitate of cultured fibroblasts. In brief, the 1068SK cells were lysed using RIPA Lysis Buffer (Upstate Biochemicals, Lake Placid, NY USA) supplemented with 1 mM PMSF. The cell lysates was then incubated with 0.3 ug/ml anti-FGF7 (Chemicon) for 1 hour then bound to Protein G beads (Sigma) for 1 hour. Beads were washed and spun down at 1,400 rpm for 1 minute in a microcentrifuge. Pelleted beads were boiled for 10 minutes at 95°C in sample buffer, centrifuged at top speed for 5 minutes and the resulting supernatant was collected for separation by SDS-PAGE on a 12% separating gel. Proteins were transferred to PVDF membrane (BioRad, Hercules, CA USA), incubated with rabbit anti-human KGF primary antibody (Chemicon) then with HRP conjugated goat anti-rabbit secondary antibody (BioRad), and visualized by Opti-4CN Detection Kit (BioRad). The negative control consisted of a lysate of a murine melanoma cell line, B16. The positive control consisted of human FGF-7 (Chemicon) as described above.

## Results

### Mast cells in fibrotic regions of tumors

**Figure 1:**
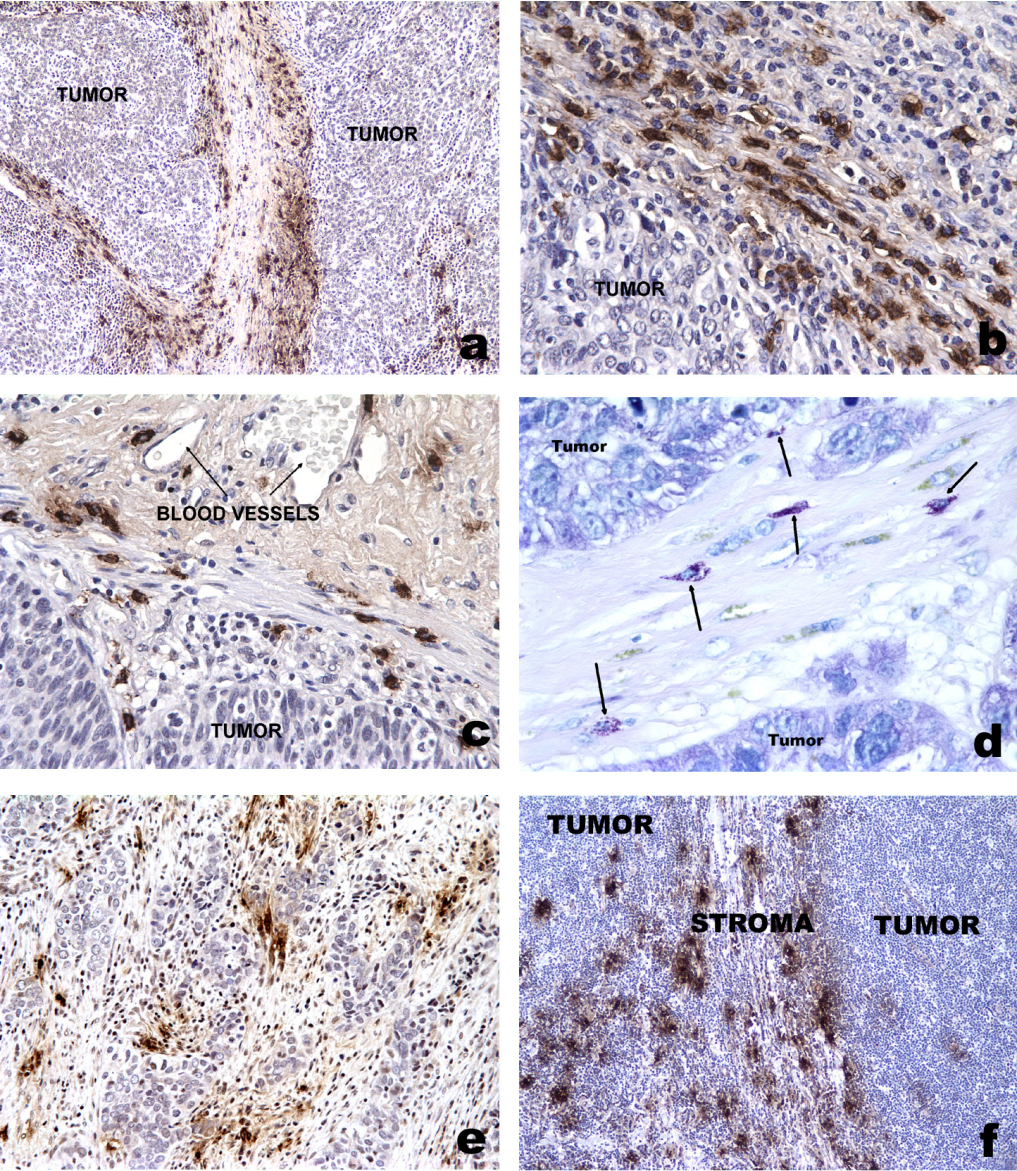
**Representative photomicrographs of mast cell infiltration in human head and neck cancer (1a), lung cancer (1b, c), ovarian cancer (1d, e), and Hodgkin's disease (1f). **In Figures a-c, e and f, the sections were immunostained for mast cell tryptase and revealed extensive evidence of mast cell degranulation (brown stain) in the fibrous tissue adjacent to nests of tumor cells and blood vessels. A Giemsa stain (1d) confirmed the presence of intact mast cells with dense purple granules embedded within fibrous stroma between nests of tumor cells. Hematoxylin counterstain (all figures, except 1d).

A summary of the results of the immunostaining for mast cell tryptase is provided in Table 1. In all of the human tumors that we examined, mast cell tryptase and intact mast cells were located predominantly at the edges of the tumors and within the fibrous stroma next to tumors (Figure 1a–e). Notably, the mast cell infiltration was significantly greater in the tumors than in the normal tissue counterparts for almost all of the tumor types that we examined. Only the Hodgkin's lymphomas and non-Hodgkin's lymphomas consistently had substantial amounts of tryptase detectable within the core of the tumors, but even in these tumors, there were more mast cells at the edges of the tumor and in the fibrous stroma subdividing the tumor mass (Table 1 and Figure 1f). The Giemsa stain confirmed that intact mast cells were almost exclusively found embedded within the fibrous stroma within tumors (Figure 1d).

### Reduction of clonogenic growth of breast cancer cells by heparin

**Figure 2:**
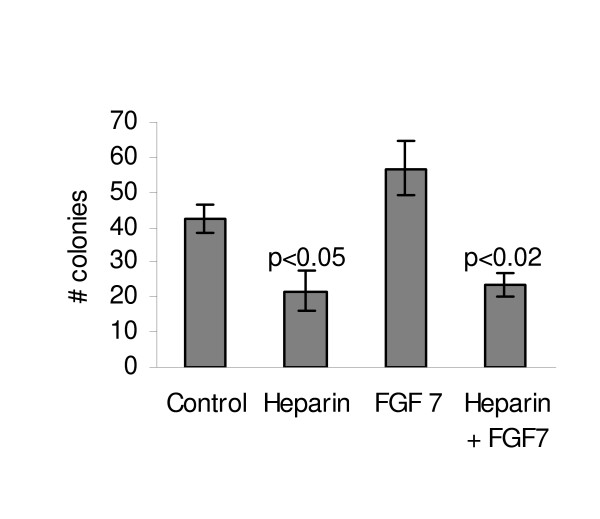
**Results of clonogenic assay of breast cancer cells co-cultured with fibroblasts in the presence or absence of heparin and/or FGF-7. **After 14 days of co-culture, the numbers of colonies containing 10 or more tumor cells were counted and averaged. Heparin significantly reduced the numbers of tumor cell colonies in the presence or absence of exogenous FGF-7. When the tumor cells were cultured in the absence of fibroblasts, only an average of 24 colonies were formed, and the heparin had no significant effect on the numbers of colonies.

The results of a representative clonogenic assay performed in triplicate with 1 unit/mL of heparin are presented in Figure 2. The experiment was repeated three times, with similar results. The consistent and notable finding was that this low dose of heparin significantly decreased the number of colonies of tumor cells in the co-culture assay with fibroblasts. The lowest concentration of heparin (0.1 units/mL) produced no detectable effect (average number of colonies = 45 ± 6, n = 3), while the higher doses of heparin (10 and 100 units/mL) produced effects very similar to 1 unit/mL (data not separately shown).

FGF-7 slightly increased the number of colonies, but this effect was neutralized by heparin. Tryptase at all of the concentrations that we tested produced no significant change in the numbers or sizes of tumor cell colonies in the co-culture assay (data not shown), despite the presence of small amounts of heparin in the tryptase solution. When the clonogenic assay was performed in the absence of fibroblasts, an average of only 23 ± 4 (n = 4) colonies were formed. Neither heparin nor FGF-7, individually (18 ± 5 colonies, n = 3) or in combination (20 ± 7 colonies, n = 3) significantly changed the clonogenic growth of breast cancer cells cultured without fibroblasts.

**Figure 3:**
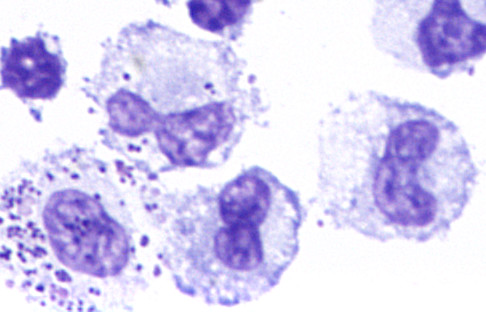
**Toluidine blue stain of cytopreparation of HMC-1 cells. **Relatively few blue granules were detected in the cultured human mast cell line, suggesting that the cultured mast cell line produced only small amounts of heparin.

Importantly, the lysate of HMC-1 cells produced no significant effect on the clonogenic growth of the breast cancer cells in the co-culture assay (average number of colonies = 24 ± 4, n = 3). A toluidine blue stain of the cultured mast cell line revealed only weak staining of small numbers of intracytoplasmic granules (Figure 3) compared to the intense staining that is normally seen in mast cell granules [11,14]. This result suggests that the HMC-1 cell line grown in our laboratory produced only small amounts of heparin.

### Reduction of size of tumor cell colonies by heparin

**Figure 4:**
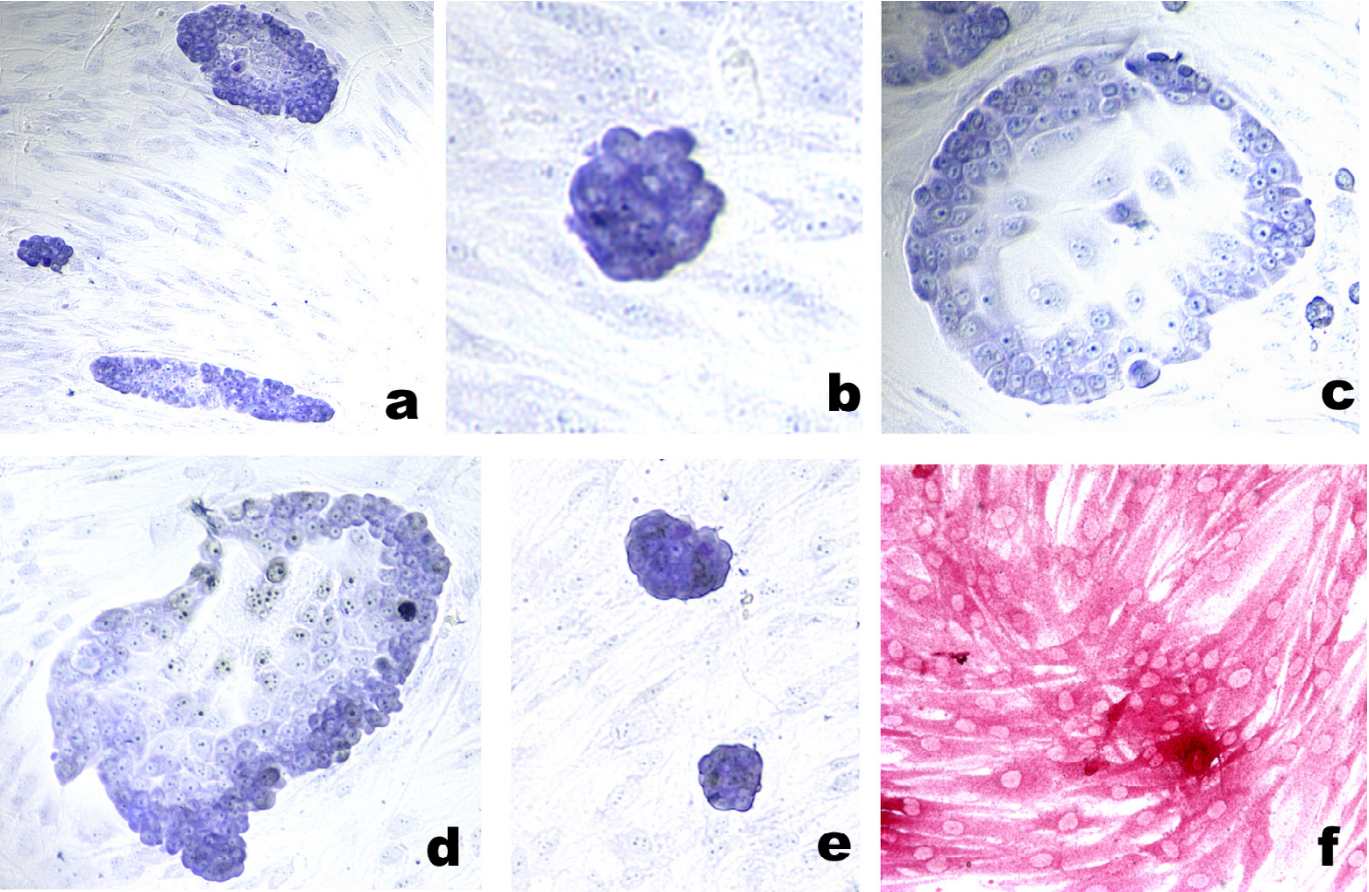
**Photomicrographs of tumor cell colonies (3a-e) and fibroblasts immunostained for FGF-7 (3f). **In the absence of heparin, breast cancer cells co-cultured on a monolayer of fibroblasts generally produced irregularly shaped large clusters of tumor cells (3a). When heparin was added to the co-culture system, the tumor cells generally formed compact clusters (3b). FGF-7 added to the co-culture system resulted in large glandular-like structures composed of a peripheral rim of compact tumor cells and a central core of plate-like epithelial cells (3c, d). Heparin completely reversed the effects of FGF-7 and produced small compact clusters of darkly staining tumor cells (3e). Immunostaining for FGF-7 (3f) confirmed the abundant presence of FGF-7 (pink stain) on the membrane of the fibroblasts. Toluidine blue stain (3a-e).

**Figure 5:**
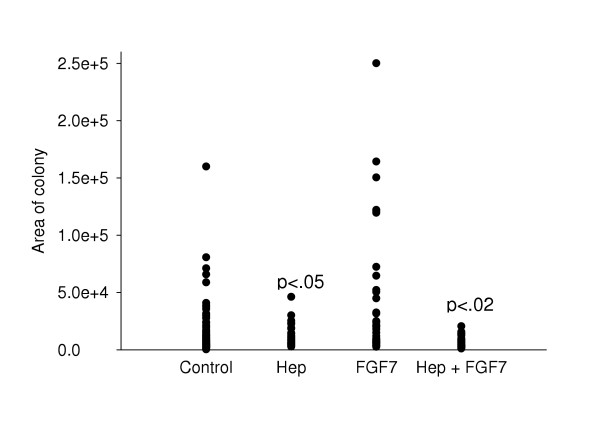
**Distribution of tumor cell colony sizes. **The average sizes of the tumor cell colonies as measured by digital image analysis were 16,428 pixels for the control; 9917 pixels for heparin alone; 35,760 pixels for FGF-7 alone; and 5551 pixels for the cells treated with heparin and FGF-7. Heparin significantly reduced the distribution of colony sizes in the presence or absence or FGF-7 as determined by a Mann-Whitney U-test.

Visual microscopic examination of the tumor cell colonies in the clonogenic co-culture assay suggested that heparin produced a striking reduction in the size of the tumor cell colonies (Figure 4). This effect appeared to be particularly pronounced when heparin was added to co-cultures supplemented with FGF-7 that, by itself, markedly increased the size of the colonies (Figures 4c, d vs 4e). A statistical analysis of the distribution of colony sizes confirmed that heparin significantly reduced the size of the tumor cell colonies relative to the control or to FGF-7 treatment (Figure 5).

### FGF-7 on membrane of cultured fibroblasts

Immunostaining studies confirmed that the fibroblasts expressed abundant FGF-7 on their cell membranes (Figure 4f). The negative control antibody and blocking buffer yielded no staining of the fibroblasts. The DNA microarray studies performed in triplicate indicated the presence of abundant mRNA coding for FGF-7 in the cultured fibroblasts (mean signal intensity 3940).

**Figure 6:**
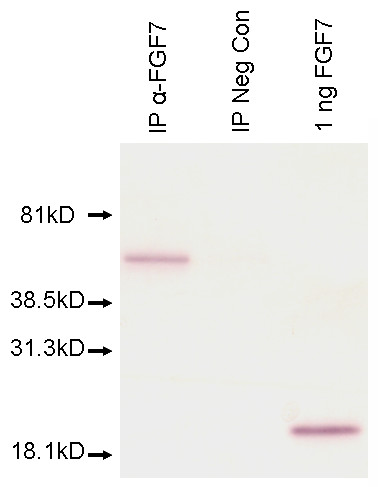
**Western blot of FGF-7 in immunoprecipitate of fibroblasts. **The polyclonal antibody recognized the positive control (approximately 22 kD; human FGF-7, lane 3) and did not bind to an immunoprecipitate of an irrelevant cell line (IP negative control, lane 2). The immunoprecipitate of the fibroblasts in lane 1 yielded a band at approximately 66 kD, which we attributed possibly to binding of FGF-7 to a heparan sulfate proteoglycan in the cell membrane.

Western blotting (Figure 6) confirmed that our anti-FGF-7 antibody specifically recognized FGF-7 (lane 3) and not an immunoprecipitate of a negative control cell line (lane 2). The immunoprecipitate of the fibroblasts (lane 1) yielded a band at approximately 66 kD, similar to the results described by Palmieri et al [18]. The higher molecular weight of the FGF-7 in the immunoprecipitate of fibroblasts is probably attributable to the presence of tightly bound heparan sulfate from the cell membrane, as described by Palmieri et al [18].

## Discussion

Our immunohistochemical studies have demonstrated that degranulating mast cells are located primarily in the peri-tumoral fibrous tissue in a wide variety of human cancers. Moreover, we have shown that heparin (a proteoglycan that is produced exclusively by mast cells) inhibits the clonogenic growth of human breast cancer cells co-cultured with normal fibroblasts but not tumor cells cultured alone. Significantly, the lysate of a human mast cell line, HMC-1, did not have any detectable effect on the clonogenic growth of the co-cultured tumor cells. We believe that this is probably due to the low levels of heparin in these cells, but we cannot exclude the possibility that some other component of mast cells may have neutralized the inhibitory effect of heparin. Similarly, various doses of tryptase had no effect on clonogenic growth. Taken together, these results provide strong evidence that mast cells can suppress the growth of tumor cells through an indirect mechanism that involves heparin and fibroblasts adjacent to the tumor cells.

This conclusion is consistent with our previous reports that depletion of endogenous heparin results in accelerated tumor growth in mice [5,14]. In specific, we previously showed that syngeneic tumor cells implanted into NDST-2 knockout mice grew faster than tumor cells implanted into wild-type mice that synthesized normal heparin [5,14]. NDST-2 knockout mice are unable to synthesize mast cell heparin and express abnormal mast cells with severely reduced amounts of histamine and mast cell proteases [11], probably because highly anionic heparin is required to stabilize the cationic compounds histamine, tryptase and chymase. Moreover, enzymatic depletion of mast cell heparin by injection of heparinase enzyme into tumor implants also accelerated tumor growth [14] and increased blood clotting within the tumors. Based on the results of the current study, we now propose that the accelerated tumor growth that we observed in heparin-depleted mice could be attributable, at least in part, to the absence of heparin-mediated inhibition of the growth-promoting interaction between fibroblasts and adjacent tumor cells.

Our findings are important because they may help to explain the well known ability of heparin and its derivatives to inhibit the growth of primary and metastatic tumors in various animal models and in humans with cancer [22-29]. Although the exact mechanism of this anti-tumor effect mediated by heparin remains uncertain, a number of possible explanations [30] have been proposed, including inhibition of thrombin and fibrin formation, immune system modulation, blockade of tumor cell adhesion to platelets, inhibition of angiogenesis, and functional inhibition of selectin-mediated cell-cell interactions leading to metastasis [31]. Heparins can also directly inhibit proliferation of various normal cell types, including smooth muscle cells and epithelial cells [reviewed in [32]]. Under some conditions, however, heparin actually stimulated the growth of epithelial cells [21]. On the other hand, only a few studies have directly evaluated the effects of heparin on the proliferation of cancer cells [also reviewed in [32]], and the results of these studies are generally inconclusive. Therefore, we propose that another possible mechanism for the anti-tumor effect of heparin observed in vivo may be the disruption of the critical, growth- promoting interactions that are known to occur between tumor cells and adjacent fibroblasts [7-9].

Although this study was not designed to explore the molecular mechanism of how heparin disrupts the interaction between fibroblasts and tumor cells, one possible explanation comes immediately to mind. Based on our findings with FGF-7, we speculate that heparin may disrupt the interaction of heparin-binding growth factors such as FGF-7 with heparan sulfate proteoglycans that are produced by fibroblasts and that act as essential co-factors for the growth factors. This explanation is consistent with our recent observation that optimal clonogenic growth of breast cancer cells in vitro requires direct physical contact between fibroblasts and breast cancer cells [13]. Moreover, previous reports from other laboratories have confirmed that human breast cancer cells are generally in close contact with fibroblasts that express abundant FGF-7 [18,19]. Finally, various heparan sulfate proteoglycans such as syndecan-1 and glypican are known to be expressed by stromal cells in cancer and to modulate the mitogenic effects of multiple heparin-binding growth factors [33,34]. In this regard, it is especially notable that heparin had no effect on FGF-7-mediated stimulation of mammary epithelial cells grown in a collagen gel matrix in the absence of fibroblasts [6]. Hence, it is reasonable to propose that heparin might interfere with the natural binding of heparin-binding growth factors to heparan sulfate proteoglycans produced by fibroblasts. Clearly, additional studies will be needed to test this hypothesis.

The possible biological and clinical significance of our in vitro experimental findings with regard to the naturally occurring mast cells that we observed around tumors remain speculative. Nevertheless, our observation that mast cells are abundantly present within the fibrous regions of tumors raises the intriguing possibility that a growth inhibitory mechanism similar to the one that we observed in our in vitro studies could also be naturally operative within tumors in vivo. The possible connection between our in vitro experiments and naturally occurring tumors is further strengthened by the reports from other laboratories that peri-tumoral fibroblasts express abundant FGF-7 [18,19] and that mast cell tryptase is intimately associated with the concurrent presence of heparin [11,12].

We acknowledge, however, that this possibility seems to be at odds with the accumulating evidence that mast cells promote rather than inhibit tumor growth [1,2]. In this regard, it should be emphasized that mast cell granules contain numerous biologically active compounds in addition to heparin, such as histamine, tryptase, and chymase. Some of these mast cell compounds and metabolites are likely to have significant effects on fibroblasts that remain to be defined. In addition, a number of other mediators from fibroblasts and mast cells could potentially influence tumor growth through a variety of mechanisms such as cyclooxygenase metabolites, heparanases, etc. The net effect on tumor growth, therefore, is likely to be the result of multiple complex interactions between the various components of mast cell granules and adjacent stromal cells such as vascular endothelium and fibroblasts. Indeed, our immunohistochemical studies also demonstrated close proximity of mast cells to peri-tumoral blood vessels as well as to fibroblasts. Consequently, it is entirely conceivable that the stimulatory effects of mast cells on angiogenesis or fibroblasts or other functions within tumors might outweigh the inhibitory effects mediated by mast cell heparin and fibroblasts.

## Conclusion

Multiple independent lines of evidence strongly suggest that heparin from mast cells can suppress tumor cell growth through an indirect mechanism involving adjacent fibroblasts. The evidence includes the localization of mast cells to fibrous regions of tumors, the ability of heparin to inhibit tumor growth in vivo and in vitro in the presence of fibroblasts, and the accelerated growth of tumors in mice that were genetically or enzymatically depleted of heparin. Thus, we propose that further molecular analysis of the interaction between fibroblasts and heparin is warranted and may lead to improved insights into how heparin mediates its anti-tumor effect and, ultimately, to improved anti-tumor therapies.

## Competing interests

MS is a paid consultant for Chemicon International (Temecula, CA) which supplied some of the reagents used in this study.

## Authors' contributions

MS conceived of the study, designed the experiments, and drafted the manuscript. EK performed the experiments and helped to draft the paper. JKC provided the ovarian cancer samples, participated in the design of this study, and critically reviewed the manuscript.

## Pre-publication history

The pre-publication history for this paper can be accessed here:



## Figures and Tables

**Table 1:** Comparison of mast cell numbers in tumors and corresponding normal tissues.

	Mean Numbers of Mast Cells/40 × Field (± 1 standard deviation)		
	
	Peri-tumoral tissue	Core of tumor	Normal tissue
Breast (n = 10)	4.9 ± 1.1	0.3 ± 0.3*	1.2 ± 0.9**
Head & neck (n = 10)	6.7 ± 3.6	0.5 ± 0.3*	2.8 ± 2.1**
Ovarian (n = 10)	3.0 ± 0.8	0.7 ± 0.6*	0.9 ± 0.8**
Lung (n = 10)	4.4 ± 1.9	1.0 ± 0.3*	2.5 ± 0.6 **
Non-Hodgkins (n = 5)	2.1 ± 1.0	1.8 ± 0.5 NS	1.0 ± 0.5 NS
Hodgkin's (n = 8)	4.3 ± 1.3	1.9 ± 0.8*	1.0 ± 0.5**

*p value < 0.05, comparing mean numbers of mast cells in core of tumor versus per-tumoral regions.

** p value < 0.05, comparing mean numbers of mast cells in normal tissue counterparts (n = 3) to peri-tumoral tissue.
